# Case Report: Presumed cerebral salt wasting syndrome in a 10-week-old German Shorthaired Pointer

**DOI:** 10.3389/fvets.2025.1553617

**Published:** 2025-03-27

**Authors:** Elizabeth Jackson, Gilad Fefer, Karen R. Muñana, Bernie D. Hansen

**Affiliations:** Department of Small Animal Clinical Sciences, College of Veterinary Medicine, North Carolina State University, Raleigh, NC, United States

**Keywords:** canine, skull fracture, hyponatremia, traumatic brain injury, hypovolemia

## Abstract

This case report describes a rare presentation of cerebral salt-wasting syndrome (CSW) in a 10-week-old German Shorthaired Pointer following a traumatic brain injury. The patient presented stuporous and tetraplegic with advanced imaging revealing a depressed skull fracture and active brain hemorrhage. Following surgical intervention, the dog exhibited persistent hypovolemia and hyponatremia prompting treatment with intravenous hypertonic saline and enteral sodium supplementation. Positive response to sodium supplementation, coupled with elevated fractional excretion of uric acid (FEUA) despite clinical improvement, supported the diagnosis of CSW. This report contributes novel insights into CSW in veterinary medicine, emphasizing the distinctive features of its presentation, diagnostic considerations, and treatment responses. The clinical utility of FEUA as a diagnostic tool is highlighted for the first time in a canine patient, providing a valuable tool for differentiation. This information enhances veterinary practitioners’ awareness, facilitating more accurate diagnoses and tailored treatment strategies for similar cases in the future.

## Introduction

Cerebral salt-wasting syndrome (CSW), also known as renal salt-wasting syndrome, is a rare condition characterized by hyponatremia and extracellular volume depletion (1, 2). Although the most common cause of CSW in humans is primary central nervous system (CNS) disorders such as trauma (3–5), this syndrome has also been reported in people without any history of CNS disease (6–8). While the exact pathogenesis of CSW remains unclear, it is believed to result from complex interactions between the CNS and the kidneys, causing excess sodium excretion. The biggest challenge in diagnosing CSW is excluding syndrome of inappropriate antidiuresis (SIAD), however numerous criteria have been proposed to differentiate between the two diseases (9–11). Although well described in the human literature, CSW in canines has only been described in one other recent case report, despite historical reference as a differential for hyponatremia (12, 13). This case report describes the diagnosis, treatment, and outcome of presumed CSW in a 10-week-old German Shorthaired Pointer puppy.

## Case description

A 10-week-old intact female German Shorthaired Pointer was presented for further evaluation of a traumatic brain injury (TBI). The dog was bitten on the head by another dog in the home, immediately becoming laterally recumbent and unresponsive. Prior to this, the dog was reportedly healthy, and up to date on her preliminary vaccines and anthelmintic series. No known familial diseases were reported prior to acquisition.

**Figure 1:**
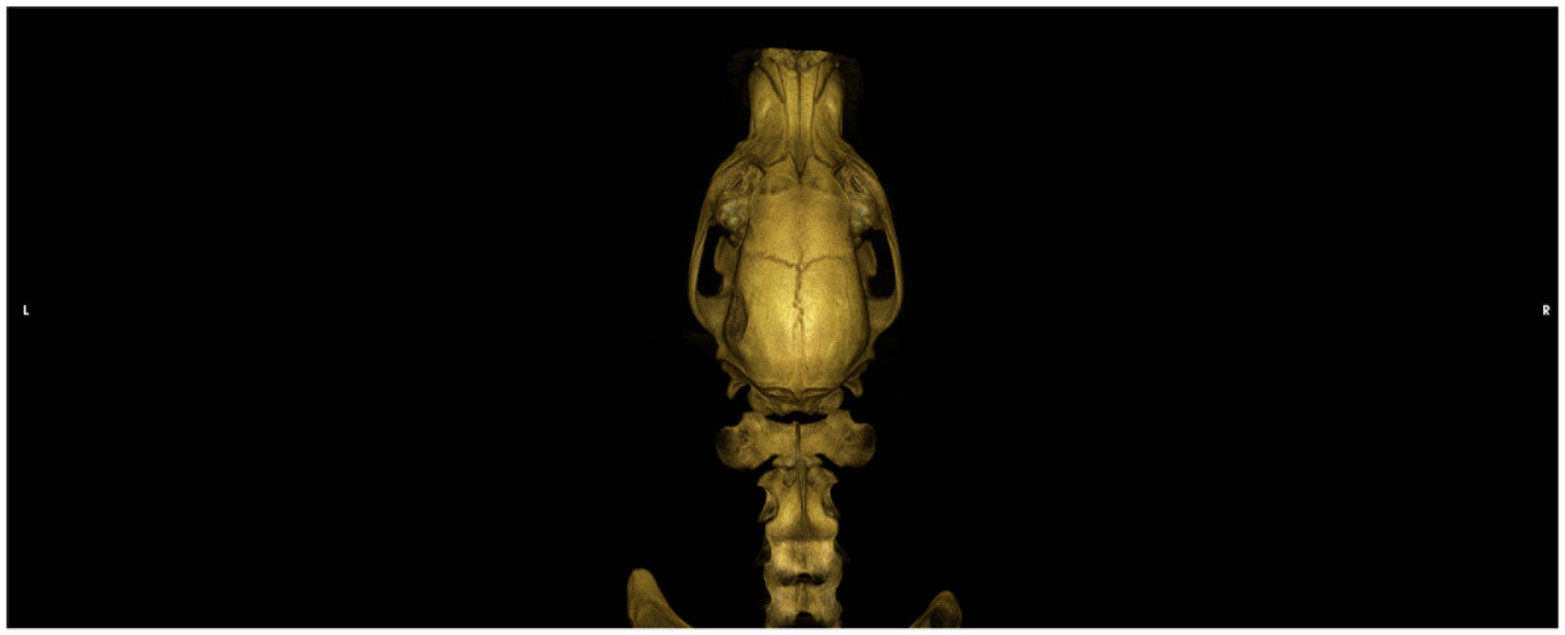
Dorsal view of a CT 3D reconstruction showing a depressed skull fracture of the left temporo-parietal bone.

Upon evaluation by the primary veterinarian immediately following the incident, the dog was stuporous, hypothermic, and tachypneic. Serum chemistry panel, CBC, serum total T4, and full body radiographs did not reveal any significant abnormalities. Serum sodium was within the reference level at 148 mmol/L. The dog received a 20 mL/kg lactated ringer’s solution (LRS) intravenous (IV) fluid bolus and supplemental oxygen. The dog was referred to a local specialty hospital for further care. Physical examination at that facility identified moderate asymmetry of the left caudal temporalis region attributed to a suspected depressed skull fracture. Neurologic examination revealed the dog to be stuporous and tetraplegic, with intact spinal reflexes in all four limbs, an absent menace response bilaterally with bilaterally miotic pupils that were minimally responsive to light, and bilateral ventral strabismus. Computed tomography (CT) of the head and cervical spine was performed, revealing a depressed left-sided temporal fracture (Figure 1). The dog received a single dose of mannitol (1 g/kg), hypertonic saline (2 mL/kg), and levetiracetam (30 mg/kg IV) followed by 60 mL/kg/day LRS IV, and a fentanyl continuous rate infusion, as well as continued oxygen supplementation. After a night at this facility with minimal neurologic improvement, the patient was then transferred to our facility approximately 48 h after the initial trauma.

**Figure 2:**
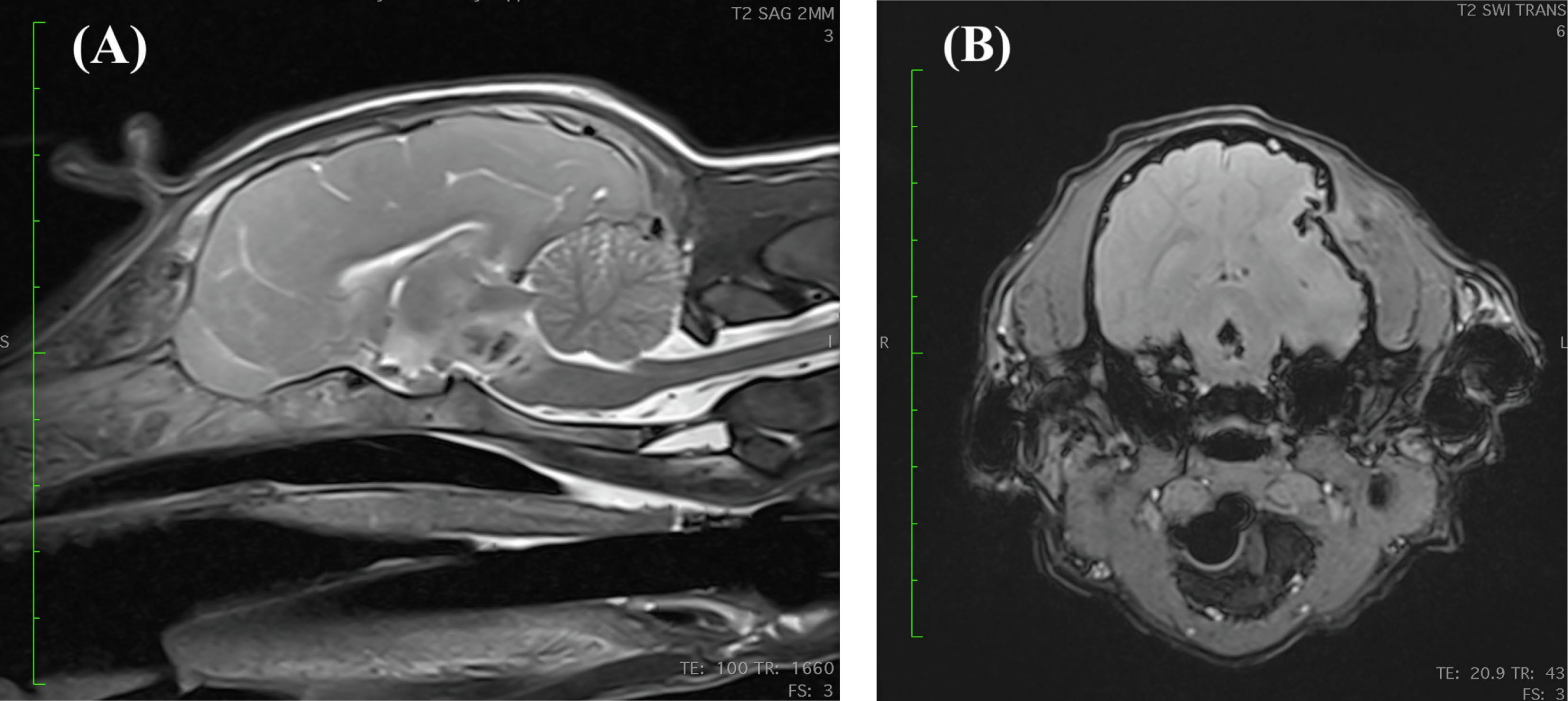
**(A)** Mid-sagittal view of a T2 weighted MRI revealing a heterogenous, potentially cavitated lesion within the pons and caudal midbrain that is centrally T2 hypointense and peripherally hyperintense. **(B)** Transverse susceptibility weighted imaging (SWI) view at the level of the caudal midbrain revealing a bone fragment being displaced medially into the cranial vault creating a concave skull defect. Additionally, there are multiple ill-defined intra-axial abnormalities with moderate susceptibility artifact at the left temporal and parietal lobes, likely representing smaller bone fragments. There is an irregularly shaped, possibly cavitated lesion within the caudal midbrain with a strong central and peripheral blooming susceptibility artifact which is highly suggestive of hemorrhage. The falx cerebri is slightly deviated toward the right side and the left lateral ventricle is compressed.

On presentation, the dog was stuporous and laterally recumbent, with appropriate perfusion parameters and mild tachypnea, weighing 7.85 kg. Neurologic examination revealed tetraplegia with absent menace response bilaterally, absent facial sensation, and miotic pupils bilaterally. The Modified Glasgow Coma Score (MGCS) upon presentation was 9. The findings suggested injury to the brainstem, as the stuporous mentation and tetraplegia would not be explained by the depressed skull fracture alone. A peripherally inserted central catheter was placed; a venous blood gas analysis obtained was normal, with serum sodium of 141 mmol/L ([137-151 mmol/L]) and adequate ventilatory parameters. No osmotic agents were administered due to concern for intracranial hemorrhage. The dog was anesthetized for magnetic resonance imaging (MRI), which confirmed a left-sided calvarial depression fracture with compression and edema of the left temporal and parietal brain cortices. Additionally, there was a cavitary lesion in the pons and midbrain most consistent with active brain hemorrhage, which was the likely cause for the stupor (Figure 2). The dog was hospitalized and treated with conservative IV fluids due to concern for cerebral edema (0.45% NaCl +0.05 mEq KCl at 50 mL/kg/day), ampicillin-sulbactam (30 mg/kg IV every 8 h), dexamethasone sodium phosphate (0.15 mg/kg IV every 24 h), pantoprazole (1.02 mg/kg IV every 12 h), ondansetron (0.52 mg/kg IV every 8 h), and acetaminophen (10 mg/kg IV every 8 h). In the first 24 h of hospitalization, the dog lost approximately 0.5 kg of body weight and appeared hypovolemic based on physical examination (persistent tachycardia, tachypnea, hypotension, and marked polyuria) and thoracic point-of-care ultrasound (POCUS) evaluation. The half strength NaCl IV fluids were replaced with LRS with physiologic KCl supplementation, and the rate of administration was incrementally increased up to 100 mL/kg/day over the next 12 h. Multiple fluid boluses were administered due to persistence of apparent hypovolemia, with no change appreciated in the dog’s volume status.

Improvement in the dog’s mentation was noted after 36 h of hospitalization, MGCS 14, and a left sided rostrotentorial craniectomy with removal of a 1.5 cm long fracture fragment to decompress the brain was performed. Pre-operative bloodwork was largely unremarkable, with a serum sodium concentration of 146 mmol/L. The patient recovered uneventfully from general anesthesia and was soon able to eat a small amount of puppy food slurry. Two days post-operatively, the dog was eating more consistently but not meeting caloric demands. A nasogastric (NG) tube was placed, and a calorically dense liquid nutrition (NutriDapt™, Canine Biologics, Denver CO) was administered to supplement voluntary food intake. The ampicillin-sulbactam was discontinued and oral amoxicillin-clavulanate (Clavamox™, Zoetis, Parsippany NJ) was initiated. Additionally, dexamethasone was discontinued in favor of oral prednisone (0.6 mg/kg/day). The IV fluids were discontinued due to financial constraints, and enteral water was administered at a rate of 20 mL/h. Later that day, the dog had a generalized tonic–clonic seizure and therapy with levetiracetam was initiated (loading dose of 60 mg/kg IV, continuation at 30 mg/kg IV every 8 h).

**Figure 3:**
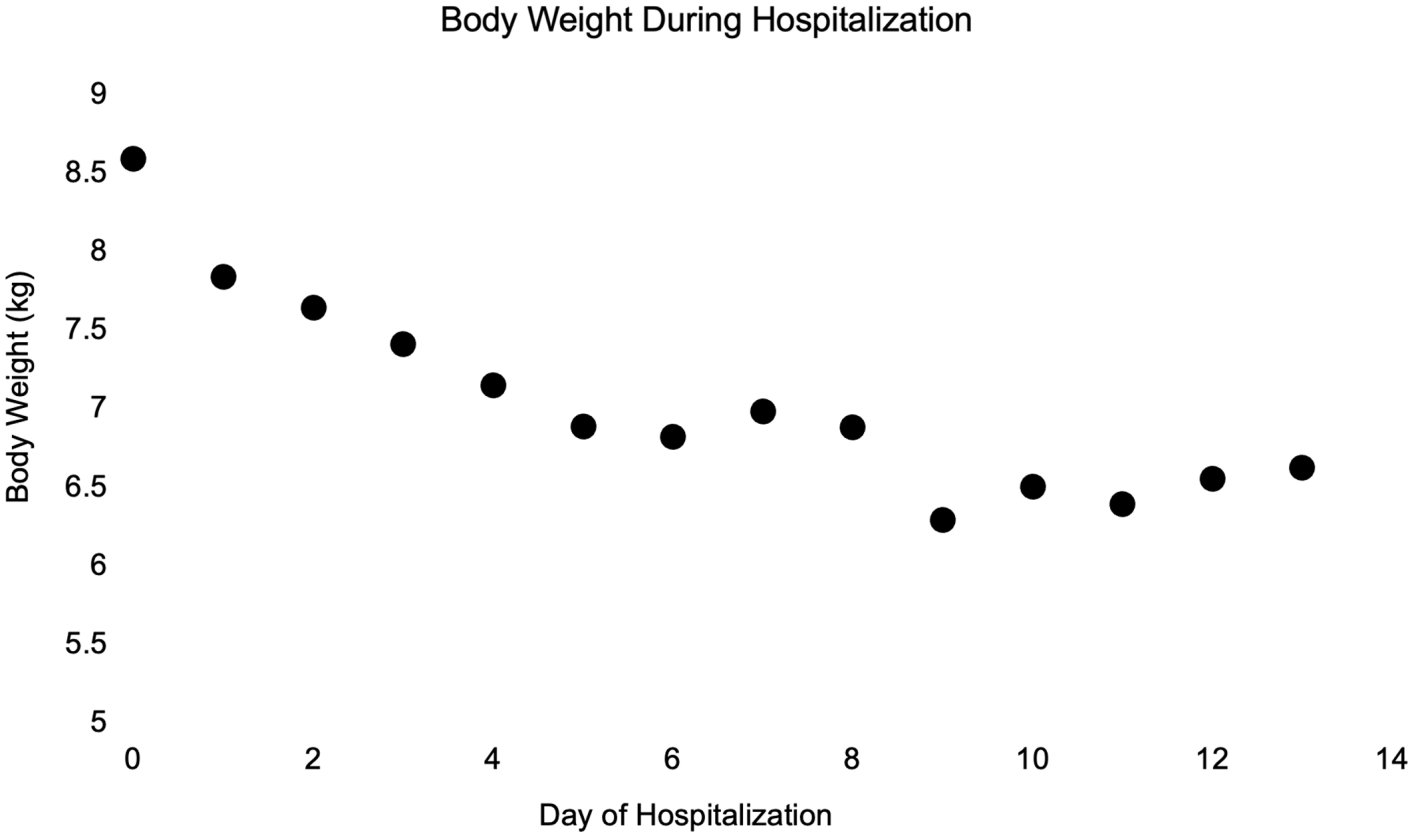
Simple dot plot depicting patient body weight in kilograms over time while hospitalized.

On day 6 of hospitalization (day 3 post-operatively), due to continued weight loss (6.80 kg) (Figure 3), and persistent physical examination findings consistent with hypovolemia despite enteral fluids and feeding, a serum biochemistry was obtained. Abnormalities included marked hyponatremia (122 mmol/L) with hypochloremia (87 mmol/L, corrected to 104 mmol/L) and normokalemia (4.5 mmol/L). Calculated serum osmolality was 258 mOsm/kg. To address the sodium deficit, with a rate not exceeding 0.5 meq/L/h, LRS supplementation (130 mEq Na/L) via the NG tube was reinstated at 60 mL/h. Serum sodium concentration was measured a few hours later, and marked hyponatremia was confirmed (119 mmol/L). The dog was administered 2 mL/kg of 7% hypertonic saline IV, with no improvement in hydration status appreciated afterward.

Despite gradual improvements in mentation and appetite, the dog remained polyuric with tacky mucous membranes and tachycardia. On day 7 of hospitalization, hyponatremia persisted (120 mmol/L) and two additional 2 mL/kg boluses of 7% hypertonic saline were administered. Approximately 8 h later, the dog remained hyponatremic (120 mmol/L) and a 7% hypertonic saline CRI IV (initially 2 mL/h for 5 h then tapered to 1.3 mL/h for 9 h) was initiated to correct the persistent volume depletion and marked sodium derangement. Administration of LRS at a rate of 60 mL/h via the NG tube was continued.

On day 8 of hospitalization, following 14 h of the hypertonic saline CRI, hyponatremia and hypochloremia had improved to 138 mmol/L and 103 mmol/L, respectively. Potassium remained within the reference interval at 4.5 mmol/L. Additional testing revealed a markedly elevated urine sodium concentration (310.5 mmol/L). Urine creatinine, urine uric acid, and serum uric acid were measured to calculate the fractional excretion of sodium (FENa 1.6% [0–0.5%]) and of uric acid (FEUA 19.71% [0–11%]). Despite the hyponatremia, the urine was moderately concentrated at 914 mOsm/kgH2O. The urine uric acid to urine creatinine ratio (uUA:uCr) was 0.20, which may further support the clinically presumed volume depletion (14). Pro-brain natriuretic peptide (proBNP) concentration measured 614 pmol/L ([0-900 pmol/L]).

The next morning (day 9), serum sodium concentration was higher than desired (157 mmol/L), and the hypertonic saline was discontinued. A CRI of enteral water (40 mL/h) via the NG tube was added to the LRS enteral supplementation. The following day, serum sodium concentration decreased to 141 mmol/L.

**Figure 4:**
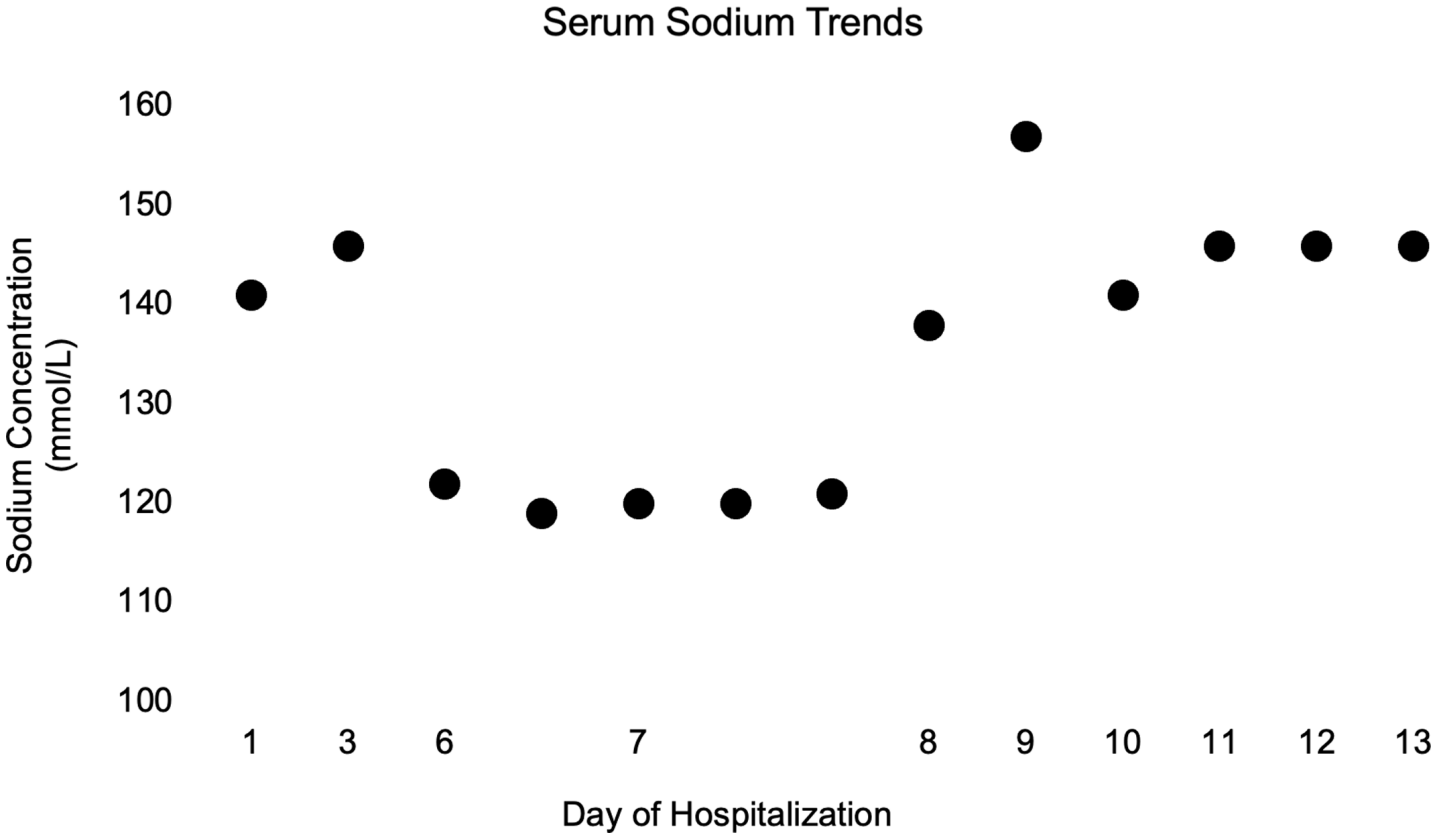
Simple dot plot depicting patient serum sodium concentrations in millimoles per liter over time while hospitalized.

To facilitate discharge from the hospital, a saltwater-food slurry supplement was devised; ½ teaspoon of salt was mixed with 1,500 mL of water to create a 35 mmol/L sodium solution. The dog was given ½ cup of this saline solution with food every 4 h, therefore supplementing approximately 4 mEq Na per feeding. The following morning (day 11), serum sodium was normalized at 146 mmol/L and enteral LRS supplementation via the NG tube was discontinued. Over the next 2 days of hospitalization, supplementation with the salt solution was reduced to ⅓ cup with every meal. Water was offered free-choice, and the saline mixture was administered with food, with no NG tube supplementation. Serum sodium concentration remained at 146 mmol/L on days 12 and 13, and the dog’s clinical hydration status markedly improved. Figure 4 depicts the complete serum sodium concentrations for the dog while hospitalized. At the time of discharge, the dog was ambulatory with significant proprioceptive ataxia in all four limbs, and occasional support necessary to prevent falls. She was blind with an absent menace bilaterally and no other cranial nerve deficits. She was discharged with prednisone (0.15 mg/kg/day) for 3 days, levetiracetam, and amoxicillin-clavulanate. The owners were instructed to continue the saline solution at ⅓ cup (~2.5 mEq Na) added to the dog’s food every 4–6 h.

**Figure 5:**
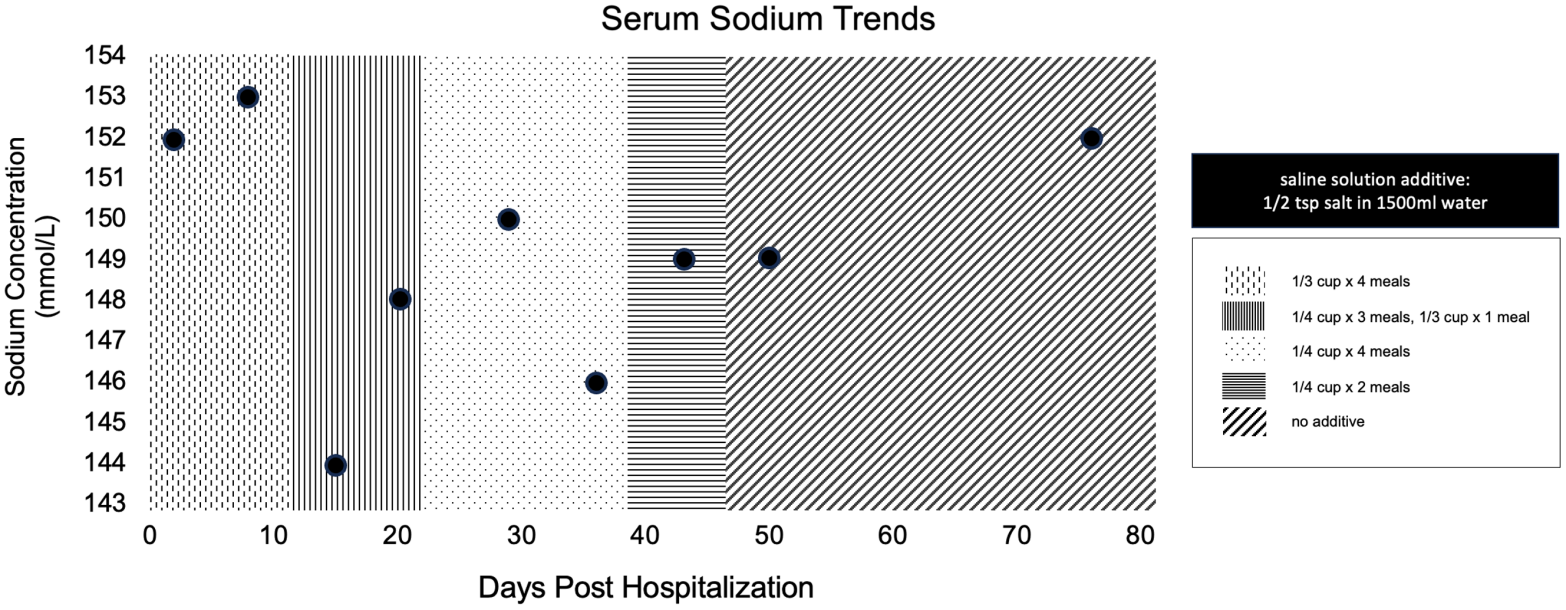
Simple dot plot depicting patient serum sodium concentrations in millimoles per liter over time at all recheck examinations post discharge. Overlaying patterns indicate amount of saline solution supplementation at the time of evaluation.

The dog was evaluated by the primary veterinarian weekly for electrolyte evaluation. Figure 5 shows the serum sodium concentrations at follow up as well as the tapering instructions for the saline solution. The saline supplementation was discontinued 6 weeks after discharge. The dog was re-evaluated by our neurology service 1 week later (8 weeks post-operatively), at which time the dog was strongly ambulatory with no obvious proprioceptive ataxia. A mildly hypermetric gait was noted, most prominent in the forelimbs. Menace response remained absent bilaterally, but the dog appeared to visually track moving objects and was able to avoid stationary obstacles. Additional testing revealed a FENa of 0.7%, FEUA of 13.4%, BNP of 697 pmol/L, and urine osmolality of 999 mOsm/kgH2O.

Approximately 5 months post hospitalization, and 4 months after discontinuing all sodium supplementation, re-evaluation of the dog’s laboratory values revealed persistent derangements. Although serum sodium concentration (146 mmol/L) and FENa (0.6%) were normal, her FEUA was 45.25%, her BNP was 758 pmol/L, and urine osmolality was 1,676 mOsm/kgH2O.

## Discussion

Two syndromes have been associated with hyponatremia in humans with brain injuries, namely the syndrome of inappropriate antidiuresis and cerebral salt wasting (12, 15). The SIAD is characterized by excessive release of antidiuretic hormone (ADH) with water retention that promotes mild hypervolemia, triggering compensatory sodium excretion in order to maintain euvolemia (16). CSW is characterized by increased renal sodium excretion causing hypovolemia, with compensatory water retention to maintain extracellular fluid volume. The treatment of each is disparate, therefore distinguishing between the two is essential; however, the clinical findings are very similar and reaching a correct diagnosis is challenging. Both syndromes are most often associated with CNS disease, resulting in hypotonic hyponatremia with an increased urine sodium concentration, and normal underlying renal, adrenal, and thyroid function (15).

CSW is clinically very similar to SIAD, but in this disorder ADH is appropriately secreted in response to volume depletion (17). Since its initial description, CSW has been most commonly reported as a sequential complication of CNS diseases in humans (3–5). More recently, some reports have described the same clinical syndrome without any concurrent CNS disease, which has prompted some to recommend renaming of the disease to renal salt wasting syndrome (8, 9). Precise definition of the syndrome is fairly controversial as the pathogenesis is poorly defined (18). Nevertheless, there is agreement that with this syndrome the kidneys are unable to conserve sodium, resulting in variable reductions in extracellular fluid volume (1).

As reviewed by Yee et al. (1) the two most widely cited theories regarding the pathogenesis of CSW implicate decreased renal sympathetic nervous system activity and increased natriuretic peptide secretion. Sympathetic innervation of the kidney plays a role in sodium absorption at the level of the proximal tubule as well as the release of renin by the juxtaglomerular cells (1, 2). Theoretically, decreased sympathetic tone would prevent the kidneys from mounting an adequate renin-aldosterone in response to decreased circulating volume. Yet, some humans with CSW are reported to have normal to elevated plasma concentrations of renin and/or aldosterone (1, 19). Renin and aldosterone concentrations were not measured in this dog.

Another theory implies excessive natriuretic peptide secretion induces natriuresis through an increased GFR and sodium excretion (1). Natriuretic peptides also have a potent effect on renal tubular function via antagonization of ADH activity and inhibition of sodium resorption (20). Identification of high serum concentrations of natriuretic peptides, particularly BNP, has been suggested as a method to differentiate CSW from SIAD. In CSW, the serum levels of BNP are elevated, whereas in SIAD, they remain within the normal range (21). However, this theory has not been easily corroborated in human CSW patients as some do not have elevated BNP concentrations, diminishing the accuracy of BNP as a diagnostic test (1). In the case described, concentrations of serum NT-proBNP, an inactive fragment of BNP with a long half-life, levels were measured at the initial onset of hyponatremia (9 days from the initial injury) as well as 1 month and 4 months after discharge; they remained at the upper end of the reference level at each measurement. One group has proposed that a proBNP cutoff of 125 pg./mL (or 458 pmol/L) can be used to distinguish between human SIAD and CSW (22). However, this was a pilot study and a definite distinguishing value of proBNP has yet to be confirmed (22, 23). The use of pro-BNP in dogs outside of cardiac diseases is extremely limited; therefore, although the high normal pro-BNP values in this case may support a diagnosis of CSW, this should be interpreted with caution.

In short, it is impossible to distinguish SIAD and CSW based on preliminary serum and urine laboratory analysis alone (24). Traditional guidelines emphasize the evaluation of patient volume status in classifying hypotonic hyponatremia and narrowing the list of differential diagnoses (18). However, the diagnostic assessment of volume status in human patients with hyponatremia has been reported to be low in sensitivity and specificity, with a wide range of subjectivity between evaluating clinicians, especially in neurologically inappropriate patients (17). Recent research has shown that diagnostic performance has improved when using an algorithm that includes both urine osmolality and urine sodium concentrations rather than overt volume status (25). The persistent elevation of FENa in this dog suggests that the hyponatremia was due to urinary excretion of sodium, but does not differentiate between etiologies of renal sodium loss.

Urine osmolality can be utilized to measure the tonicity of urine, revealing both solute output and concurrent ADH influence. A low urine osmolality indicates suppression of ADH activity, which is a normal response to low serum osmolality and allows excretion of excess free water (12, 18). Contrarily, an elevated urine osmolality indicates a response to ADH that impairs urine dilution. The dominant mechanism underlying hypotonic hyponatremia is impaired urinary dilution due to increased ADH activity, which can be physiologically appropriate or inappropriate depending on the patient’s volume status (18). In the case described, the dog consistently had a moderate elevation in urine osmolality, indicating an ADH dependent cause of hyponatremia but did not further distinguish the underlying etiology.

Recently, fractional excretion of uric acid (FEUA) has been used in differentiation of human SIAD from CSW (9). Uric acid is a product of purine metabolism that is freely filtered at the renal glomerulus and co-transported with sodium at the proximal tubule. The FEUA quantifies the percent excretion of the filtered load of uric acid across the glomerulus (7, 9). Multiple tubular uric acid transporters, have been identified that couple uric acid reabsorption to that of sodium (9).

A normal value for FEUA is <11% in humans, whereas patients with untreated SIAD and CSW have consistent elevations of FEUA >11% at the onset of hyponatremia (7, 9). After correction of the hyponatremia, the FEUA normalizes (<11%) in patients with SIAD but will remain persistently elevated (>11%) in patients with CSW (7). Thus, FEUA has been proposed as a more quantifiable measure to differentiate SIAD from CSW. In the case described, the FEUA was persistently elevated despite the resolution of hyponatremia and clinical volume status, as well as normalization of uUA:uCr at subsequent recheck evaluations.

The dog described in this report had markedly increased urinary sodium excretion occurring concurrently with findings suggesting hypovolemia, consistent with a diagnosis of CSW. The dog’s hyponatremia rapidly corrected after initiation of a hypertonic saline CRI. The positive response (i.e., increased mucous membrane moisture, skin turgor, normalization of heartrate and blood pressure, and reduction in weight loss) to sodium supplementation and continued isotonic enteral supplementation also supports the diagnosis of CSW (2).

There are several limitations to this case report. First, the patient was never directly screened for hypoadrenocorticism. Hypoadrenocorticism may also present with marked hyponatremia and either euvolemia or hypovolemia depending on the severity of the clinical signs. However, the dog’s hyponatremia resolved without mineralocorticoid supplementation. Another limitation to this report is the relative subjectivity of evaluating the dog’s volume status. The traditional guidelines for distinguishing SIAD from CSW rely heavily on whether the patient is euvolemic or hypovolemic, a challenging prospect unless physical examination changes are pronounced. The uUA:uCr was also quantified in this case to aid in volume status estimation.

Furthermore, the fractional excretion measurements utilized are only evaluating a single point in time. These values can be influenced postprandially, although the dog being fed the same diet after discharge will limit this variability. Ideally, pooled urine samples collected over 24 h should be utilized to acquire a more accurate fractional excretion of sodium, however this would not have been feasible to perform on an outpatient basis during recheck examinations. Finally, the interpretation of FEUA was made based solely on reference ranges in people; further investigation is required regarding normal FEUA in domestic animals.

In conclusion, we have described a juvenile dog with persistent hypotonic hyponatremia and clinical findings consistent with CSW secondary to a TBI. The dog lost weight and appeared dehydrated while developing marked polyuria and remained severely hyponatremic and hypovolemic despite replacement fluid administration. The hyponatremia resolved only with administration of hypertonic sodium chloride and oral sodium supplementation. Urine osmolality and urine sodium concentrations were markedly elevated in all measurements, suggesting an ADH dependent process and renal specific sodium loss. The elevated FENa (>0.5%) despite clinical evidence of hypovolemia and an elevated uUA:uCr also supports these assumptions (26). Subsequent testing revealed an increased FEUA, which remained persistently elevated despite the resolution of her hyponatremia, which aided in the differentiation of CSW from SIAD. This additional diagnostic evaluation expands the knowledge base on this condition in canine patients as previously described in the recent report from Chromiak et al. (13).

## Data Availability

The original contributions presented in the study are included in the article/supplementary material, further inquiries can be directed to the corresponding author.
